# A loopy view of telomere evolution

**DOI:** 10.3389/fgene.2015.00321

**Published:** 2015-10-20

**Authors:** Titia de Lange

**Affiliations:** Laboratory for Cell Biology and Genetics, The Rockefeller University, New York, NY, USA

**Keywords:** telomere, telomerase, Group II intron, replication, DNA damage, eukaryote

## Abstract

About a decade ago, I proposed that t-loops, the lariat structures adopted by many eukaryotic telomeres, could explain how the transition from circular to linear chromosomes was successfully negotiated by early eukaryotes. Here I reconsider this loopy hypothesis in the context of the idea that eukaryotes evolved through a period of genome invasion by Group II introns.

## Why Linear Chromosomes?

Before the linear chromosomes of eukaryotes emerged ∼1 Gy ago, circular chromosomes had been successfully used for 2 Gy and they continue to predominate in the most common organisms on this planet (eubacteria and archaea). What were the disadvantages of circular chromosomes that could have ensured the supremacy of an incipient eukaryote with linear chromosomes?

It has been suggested that the answer lies in the first division of meiosis (Ishikawa and Naito, 1999). Meiosis may have first evolved as a mechanism to correct polyploidy arising from genome segregation mistakes. Furthermore, the counterpart of meiosis, the syngamic fusion of haploid cells to form diploids may have been advantageous to survive famine as well as providing greater resistance to the highly mutagenic environment that existed 1 Gy ago. It was argued that switching between diploid and haploid states poses a problem for organisms with circular chromosomes. In the reductional division of meiosis, the homologous chromosomes are held together by their chiasmata where recombination has generated a crossover between homologous chromatids. A single meiotic crossover (or any uneven number of crossovers) generates a dicentric circle, which will lead to non-disjunction of the homologs. As this problem is circumvented with linear chromosomes, linearization may have provided a selective advantage.

This argument ignores systems like the bacterial XerD/C resolution machinery, which efficiently cuts dimeric circular chromosomes at specific dif sites (Barre et al., 2001). A similar system could have been used to resolve dimeric circles in the meiosis of early eukaryotes. Below I propose that linear chromosomes arose as the consequence of the invasion of a circular genome with repeat sequences. Once formed, linear chromosomes may have had advantages under certain circumstances but their raison d’etre, I argue, is found in the way linear DNAs with repetitive sequences at their termini escape re-circularization through ligation.

## T-Loops as a Primordial Telomere System

**FIGURE 1:**
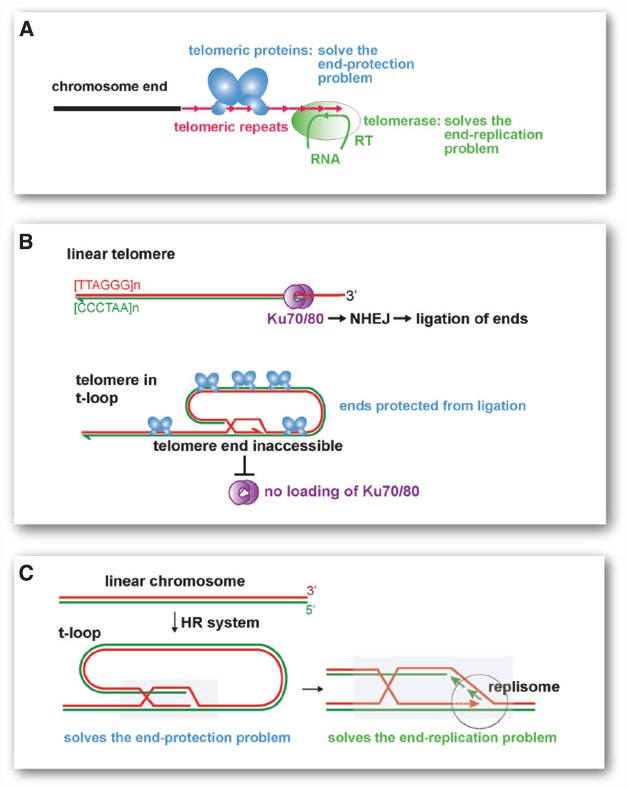
**Modern telomeres and their proposed t-loop precursor. (A)** Current telomeres require a telomerase that synthesizes the telomeric repeats and counteracts the end-replication problem. They also require telomere specific proteins that recognize the telomerase products at chromosome ends and protect the ends from the DNA damage response (solving the end-protection problem). **(B)** Mammalian telomeres form t-loops, which sequester the telomere end and prevent ligation by NHEJ. Telomeric proteins (blue, e.g., TRF2) are needed to form the t-loop structure. Telomeric proteins also protect telomeres from other DNA repair pathways and prevent the activation of the DNA damage signaling pathways (not shown). **(C)** The t-loop based primordial telomere. The proposed precursor to modern telomeres is a t-loop structure as depicted. The critical aspect of the t-loop is the strand-invasion (mediated by homologous recombination factors) of the telomere end into a repeated homologous sequence (gray box). The invaded repeat could either be close to the end or chromosome-internal. Any repetitive sequence of sufficient length to allow homologous recombination can fulfill this function. Although the strand-invasion would require a 3′ overhang, recruitment of a replisome and DNA synthesis would generate a structure lacking single-stranded DNA (shown on the left). The strand-invasion of the end blocks NHEJ and ssDNA recognition systems (e.g., SOS response), thus solving the end-protection problem. When the terminal sequences is extended by DNA replication, the end-replication problem is solved (right).

The modern eukaryotic telomere is a complex system of two critical components (Figure 1A). The maintenance of telomeres requires the telomerase reverse transcriptase with its associated RNA template, which dictates the sequence repeats at the chromosome ends. This system ensures the presence of telomeric repeats despite their constant erosion with conventional replication [the “end-replication problem” (Watson, 1972; Olovnikov, 1973)]. Furthermore, the action of telomerase endows every chromosome end with binding sites for sequence specific telomere proteins. It is the presence of such telomeric proteins that protect the telomeres from being detected as sites of DNA damage, thus solving the “end-protection problem” (de Lange, 2009). Without telomeric proteins, the telomeric repeats do nothing to repress the DNA damage response and chromosome ends become substrates for DNA repair. *Vice versa*, without the telomerase-derived telomeric repeats, the telomeric proteins are incapable of preventing genomic mayhem. It seems unlikely that both components of the telomere system, telomerase with its RNA template on the one hand and the sequence specific binding proteins that recognize the DNA version of this template sequence on the other, arose simultaneously. Of course, intermediate steps can be envisaged. For instance, the earliest telomerase RNA may have dictated sequences that happened to interact with a pre-existing DNA binding protein capable of some protection. But a much simpler scenario is suggested by the t-loop structure, the lariats found at present-day telomeres that play a critical role in telomere protection.

T-loops are double-stranded looped structures that are formed through the strand-invasion of the telomere terminus into the telomeric repeats (Figure 1B). Telomeres generally have a 3′ overhang that facilitates the formation of t-loops and modern telomeres contain specific proteins that are critical for the formation/stabilization of this structure. In mammalian cells, t-loops block DNA repair by non-homologous end joining (NHEJ; see Figure 1B), which would generate end-to-end fused chromosomes. T-loops also represent a powerful mechanism for hiding the chromosome end from the ATM kinase-dependent DNA damage response, which would result in cell cycle arrest. Similarly, in the incipient eukaryote, a modified version of the t-loop structure could have protected the ends from resident nuclease and ligases (Figure 1C). Furthermore, if the structure at the base of the t-loop lacked single-stranded DNA, the chromosome ends would not have activated the bacterial SOS response, which detects DNA damage when ssDNA is formed (Baharoglu and Mazel, 2014). Although strand-invasion would require a single-stranded overhang and thus create a single stranded D (displacement) loop, a t-loop lacking ssDNA can be generated if the D loop is converted into double-stranded DNA by fill-in DNA synthesis (see below, Figure 1C).

As discussed in detail previously (de Lange, 2004), t-loops not only solve the end-protection problem, they also provide a mechanism for extending the terminal sequences without the aid of telomerase (Figure 1C). The structure at the base of the t-loop is identical to the structure of a replication fork. *De novo* recruitment of replication enzymes could ensure that the end is extended, solving the end-replication problem. These steps would not require evolution of new factors because the machinery that mediates replication restart events in bacteria is able to execute them.

The solution to the end-replication problem afforded by the t-loop structure is related to the telomere maintenance systems observed under certain circumstances in present-day eukaryotes. An example is the alternative lengthening of telomeres (ALT) pathway for telomere maintenance, which is a pathway active in a subset of human cancers that maintains telomeres by homologous recombination (HR). Although the exact mechanism of telomere elongation by ALT is not known, one of the proposed mechanisms involves extension of telomeres in the t-loop configuration (see de Lange, 2004).

## Group II Introns and the Inevitability of Linear Chromosomes

As outlined above, the incipient eukaryotes could have had stable linear chromosomes without the need for telomerase or telomere specific proteins. The only necessity would have been terminal sequences that are homologous to more internal sequences so that the critical strand-invasion event can take place (Figure 1C). There is no need for an array of repeats at the ends. The ends could invade an internal copy of the repeat with exactly the same outcome of protection and replicative extension of the termini. But where did these repeats come from?

**FIGURE 2:**
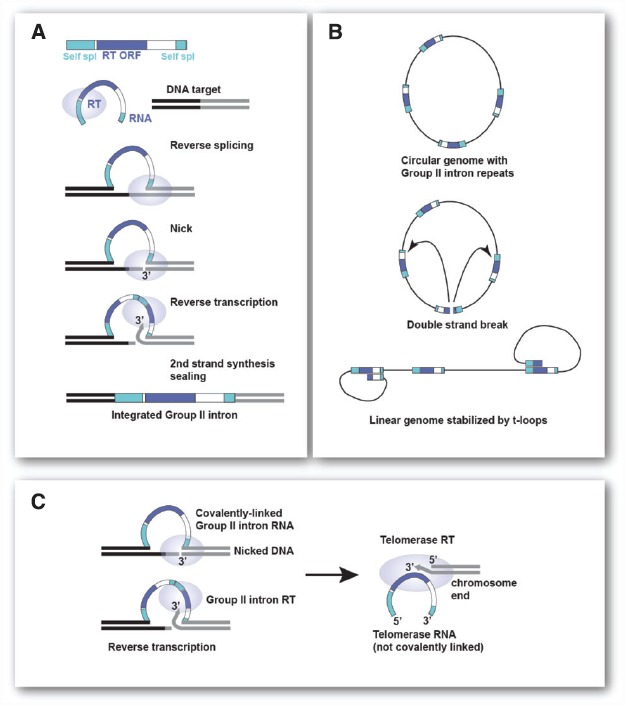
**Group II introns and their role in chromosome linearization. (A)** Steps involved in insertion of mobile Group II intron elements by target-primed reverse transcription. The Group II RNP recognizes the DNA target site and reverse splices into the top strand. The Group II endonuclease cleaves the bottom strand and the free 3′ OH is the primer for reverse-transcription. Host repair activities, which vary across organisms, complete the process (see Lambowitz and Belfort, 2015, for details on Group II introns). **(B)** Generation of a stable linear chromosome from a circular genome containing multiple Group II introns. A double strand break in one of the Group II introns can give rise to a linear chromosome that is stabilized by strand-invasion of the terminal Group II sequences into internal Group II introns. Such t-loop ends will be protected from ligation and allow extension of the terminal sequence (see Figure 1C). **(C)** Changes in Group II intron RT needed for telomerase function. Left: Reverse transcription of the Group II intron RNA that has been self-spliced (reverse reaction) into the genomic DNA. RT uses a 3′ end generated by endonucleolytic cleavage to prime reverse transcription of the covalently attached Group II intron RNA. Right: To function as a telomerase, the Group II RT has to be able to use the 3′ end of a chromosome to prime reverse transcription of a non-covalent RNA template bound to the enzyme.

Although any repeat element of sufficient length and present at the required copy number would in principle provide circular genomes with the same high chance of becoming linear, I propose that mobile Group II self-splicing elements (Group II introns) are a good candidate for the repeats that led to chromosome linearization. Group II introns are the proposed ancestors of introns and non-LTR retrotransposons. These elements use reverse splicing and reverse transcription to efficiently integrate into specific DNA target sites (see Figure 2A). They can also spread through the genome by a similar, but less efficient reaction at ectopic sites.

Cavalier-Smith (1991) and Koonin (2006) have argued that the phagocytosis of an *α*-proteobacterial cell by archaeal (or actinobacterial) eukaryotic precursor could have been accompanied by massive invasion of Group II introns (Martin and Koonin, 2006). The Group II introns residing in the genome of the ingested future mitochondrion are proposed to have colonized the host genome resulting in a large number of repetitive elements (see Figure 2A, for schematic of Mobile Group II introns). The insertion of Group II introns into coding regions could have provided the selective pressure for the invention of the nucleus as a compartment where introns can be removed from pre-mRNAs before they are used by ribosomes (Koonin, 2006; Martin and Koonin, 2006; but see Cavalier-Smith, 2010, for a dissenting opinion). The removal of the Group II introns may have initially involved protein assisted self-splicing with protein-dependent splicing evolving later. The invasion of Group II introns may have also led to nonsense-mediated decay as a way to remove intron-bearing transcripts from ribosomes but generation of a nuclear envelope combined with a system that links mRNA transport to the completion of splicing is a more definitive solution (Koonin, 2006; Martin and Koonin, 2006).

I propose that accumulation of Group II introns in the genome could also have generated the condition under which linear chromosomes became inevitable. Consider a future eukaryote with a circular genome full of Group II introns (Figure 2B). If a double-strand break occurred in one of these repeats, the bacterial DNA repair machinery would have acted in one of two ways. Either the ends would be ligated back together by some form of NHEJ or the ends would have been processed by the HR machinery of the host. The strand-invasion by HR would have had to take place in other copies of Group II introns, since they would be the only homologous target. The initiation of recombination would have generated a terminal loop, a t-loop, of variable size and sequence composition (Figure 2B). A linear chromosome containing such t-loops at each end would be impervious to re-ligation and terminal sequence attrition would be counteracted by extension of the ends using the mechanism shown in Figure 1. Thus, once formed, such a linear chromosome would be stable. The chance of this scenario playing out is greater as the number of repeats increases. Once a linear with t-looped ends is formed, the path back to a circular genome is difficult because the ends are protected. During DNA replication of the t-loop, the ends would be free to undergo ligation thus reforming a circular chromosome. But this would only happen if the replication forks synchronously dislodged the t-loops. Even if re-circularization happened, there would be a good chance of another double strand break (DSB) occurring in a Group II intron leading again to a linear state. Thus, the earliest eukaryotic chromosomes may have existed predominantly as linears that occasionally were converted to a circular state.

## From Dispersed Group II Introns to Telomere Specific Repeats

After a period of semi-stable linear chromosomes, a more permanent linear state would have required the gradual evolution toward the system used by modern telomeres. Two major steps are needed for this to happen. First, the telomerase system would have to evolve. The telomerase reverse transcriptase is likely derived from the reverse transcriptase (RT) of Group II introns (Nakamura and Cech, 1998; Dlakić and Mushegian, 2011). In order to become a true telomerase, the Group II RT would have had to gain the ability to use the 3′ end of a chromosome as a primer for reverse transcription of its associated RNA. Furthermore, it would have needed to use its RNA as a template even though it is not covalently linked to the target site (see Figure 2C). Once these modifications were made to one of the Group II intron RTs, the Group II intron sequences that became the future telomerase RNA could have evolved to cooperate with this enzyme. Thus, one Group II intron would encode the telomerase RT and another would encode the telomerase RNA. Both can now evolve into new genes that execute the terminal extension efficiently and repeatedly without the encoding genes being burdened by the requirements for self-splicing and other Group II intron functions. The RNA component can now change to 1. associate only with the telomerase RT; 2. specify a short sequence as a template for terminal sequence addition rather than the whole RNA; and 3. enable synthesis of an array of the same short repeats at every chromosome end.

The resulting system would have created linear chromosomes with arrays of short repeats that are telomere specific and no longer have homology to Group II introns. At this stage, the t-loops will only form within the telomeric repeat array since this is the only homologous sequence available in the genome.

Once all chromosome ends have the same sequence, the incipient eukaryote could evolve proteins that recognize this sequence. These early telomeric proteins are likely to be selected for their ability to mediate the t-loop structure since this was the critical aspect of telomere protection. They may also have had the ability to bind to the telomerase RT, thereby ensuring the maintenance of the telomeric repeats. These features are still present in modern telomeres. For instance, the telomeric repeat binding factor 2 (TRF2) component of the mammalian telomeric complex (shelterin) enables t-loop formation whereas other factors in shelterin recruit telomerase (Nandakumar et al., 2012; Zhong et al., 2012; Doksani et al., 2013; Sexton et al., 2014).

## Why Such Elaborate Telomeres?

The scenario sketched above raised the question why telomeres became so elaborate. Why not stick with the simple t-loop mode? Why have telomerase and a host of telomeric proteins? The same question could be asked about intron splicing which evolved from simple self-splicing based on RNA catalysis to elaborate spliceosomal complexes with a myriad of RNA and protein components. In part, the answer must be that most processes in eukaryotes generally evolve toward complexity, presumably because complexity provides more regulatory opportunities and perhaps also because there is no selective pressure to enforce simplicity.

With regard to telomeres, there is an additional consideration. Telomeres need to adapt to the DNA repair pathways and DNA damage signaling pathways that evolve in their host cells. These pathways have become increasingly complex and more varied. In response, telomeres have attained additional bells and whistles to help protect chromosome ends from these pathways (de Lange, 2009). In contrast, the end-replication problem has remained the same. As a result, the way telomeres deal with the end-protection problem and the protein complexes used for this task are highly variable while telomerase has been conserved.

### Conflict of Interest Statement

The author declares that the research was conducted in the absence of any commercial or financial relationships that could be construed as a potential conflict of interest.
